# Exploring the Relationship Between Spirometry-Defined COPD and Periodontitis Among US Adults

**DOI:** 10.3390/healthcare14213727

**Published:** 2026-11-03

**Authors:** Suha Alsaidi, Myrna Douna, Ethan Raghunanan, Diego Bodden, Babu Mandhidi, Meghan Keskar, Giang T. Vu, Bert Little

**Affiliations:** 1 School of Global Health Management and Informatics, University of Central Florida, Orlando, FL 32801, USAdiego.bodden@ucf.edu (D.B.);; 2 Orange Blossom Family Health, Orlando, FL 32805, USA; 3 School of Public Health and Information Sciences, University of Louisville, Louisville, KY 40202, USA

**Keywords:** pulmonary disease, chronic obstructive, periodontitis, spirometry, nutrition surveys, cross-sectional studies, United States

## Abstract

**Background**: Chronic obstructive pulmonary disease (COPD) and periodontitis are both chronic inflammatory conditions that share common risk factors and biological pathways. This cross-sectional study analyzed the relationship between spirometry-defined COPD and periodontitis among US adults using data from the National Health and Nutrition Examination Survey (NHANES). **Methods**: Data from the 2009–2012 NHANES cycles were used. The study sample included 4337 adults aged 30–79 years. COPD was defined as a post-bronchodilator FEV1/FVC ratio below the lower limit of normal. Periodontitis was measured by clinical examination using the 2012 case definitions for population-based surveillance of periodontitis by the Centers for Disease Control and Prevention (CDC) and the American Academy of Periodontology (AAP). Multiple weighted logistic regression was used to analyze the relationship between periodontitis and COPD, controlling for diabetes, smoking, income, education, sex, race, and age. **Results**: Adults with COPD had significantly higher adjusted odds of periodontitis (aOR = 1.99, 95% CI: 1.16–3.41, *p* = 0.015). Diabetes was also associated with higher odds (aOR = 1.65, 95% CI: 1.15–2.36, *p* < 0.01). Current or former smokers, those with lower income, and those aged ≥ 65 years had higher odds of periodontitis, whereas being female and having at least some college education were associated with lower odds. Black, Hispanic, and other race/ethnicity adults had higher odds than non-Hispanic White adults. **Conclusions**: Spirometry-defined COPD was associated with periodontitis among US adults. These findings support the need for more integrated approaches to managing chronic respiratory disease and dental care, especially in at-risk populations.

## 1. Introduction

Chronic obstructive pulmonary disease (COPD) affects more than 14 million US adults. Chronic lower respiratory diseases, of which COPD is the main component, are the fifth leading cause of death in the United States, resulting in about 146,000 deaths annually [1,2]. This chronic lung disease, marked by inflammation, may also affect periodontal health. Current evidence suggests that periodontitis is not only associated with respiratory diseases such as COPD but also with several other systemic conditions [3]. Chronic inflammatory disorders, including diabetes mellitus and cardiovascular disease, have shown a shared relationship with periodontal disease [4,5]. Common oral health findings among people with COPD include more dental plaque, deeper periodontal pockets, greater clinical attachment loss, and fewer remaining teeth compared with adults without COPD. Oral pathogens can be aspirated into the lower airways [6,7]. Several biologically plausible mechanisms may explain the association between COPD and periodontitis. Both diseases are characterized by chronic inflammation and dysregulated immune responses, particularly involving neutrophils and macrophages [8]. In COPD, excessive neutrophilic inflammation contributes to tissue destruction, airway remodeling, and impaired innate immunologic defense [9]. Similarly, periodontitis is driven by a sustained inflammatory response to disrupted oral biofilms, leading to connective tissue breakdown and bone resorption [10]. Shared inflammatory mediators, including pro-inflammatory cytokines and reactive oxygen species (ROS), may amplify disease processes at both the oral and pulmonary sites [11].

Periodontitis is a major oral health concern in the US, influenced by advanced age, tobacco smoking, lower income, less than high school education, and systemic diseases (e.g., diabetes) [12]. Characterized by different levels of inflammation and bone loss, periodontitis affects about 42% of dentate US adults who are 30 years of age or older [13]. It is projected that periodontitis will remain a stable burden in the United States population through 2050 [14]. Several observational studies and systematic reviews have reported associations between periodontitis and respiratory conditions such as asthma, pneumonia, and COPD [6]. Recent population-based surveys suggest that individuals with COPD are more likely to have periodontitis than those without COPD, even after accounting for shared risk factors such as smoking and age [15]. A recent analysis of the National Health and Nutrition Examination Survey (NHANES) by Zhang et al. also reported an association between periodontitis and COPD, which was defined by a fixed ratio of forced expiratory volume in 1 s to forced vital capacity (FEV1/FVC) below 0.70 in 2009–2012 and by self-report in 2013–2014 [16].

Despite this possible link, few national studies have examined the association. Moreover, many existing studies are limited by small sample sizes [6], non-representative populations [6], reliance on self-reported COPD diagnoses [15], or inconsistent definitions of periodontitis [6], underscoring the need for updated analyses using nationally representative data and standardized periodontal case definitions. In addition, studies that used spirometry, including a recent NHANES analysis [16], defined COPD by a fixed FEV1/FVC ratio below 0.70, which does not account for the normal age-related decline in lung function, whereas a clinical diagnosis of COPD requires airflow limitation that persists after bronchodilation [17,18]. Therefore, we aimed to evaluate the association between post-bronchodilator spirometry-defined COPD and periodontitis among US adults using nationally representative NHANES data to address gaps in prior population-based evidence. We hypothesized that adults with spirometry-defined COPD would have higher odds of periodontitis than adults without COPD, independent of shared risk factors such as smoking, age, and diabetes.

## 2. Methods

### 2.1. Data Source

This study analyzed publicly available data from the 2009–2010 and 2011–2012 cycles of the National Health and Nutrition Examination Survey (NHANES), the most recent cycles in which both a full-mouth periodontal examination and spirometry were performed. NHANES is conducted by the Centers for Disease Control and Prevention (CDC) and is designed to assess the health and nutritional status of adults and children in the United States. The survey employs a multistage probability sampling design, allowing findings to be generalized to the US civilian, non-institutionalized population. Data are collected through in-home interviews, which obtain demographic, socioeconomic, and health information, followed by standardized physical and dental examinations, laboratory tests, and spirometry performed by trained staff in mobile examination centers [19,20].

### 2.2. Study Population

**Figure 1:**
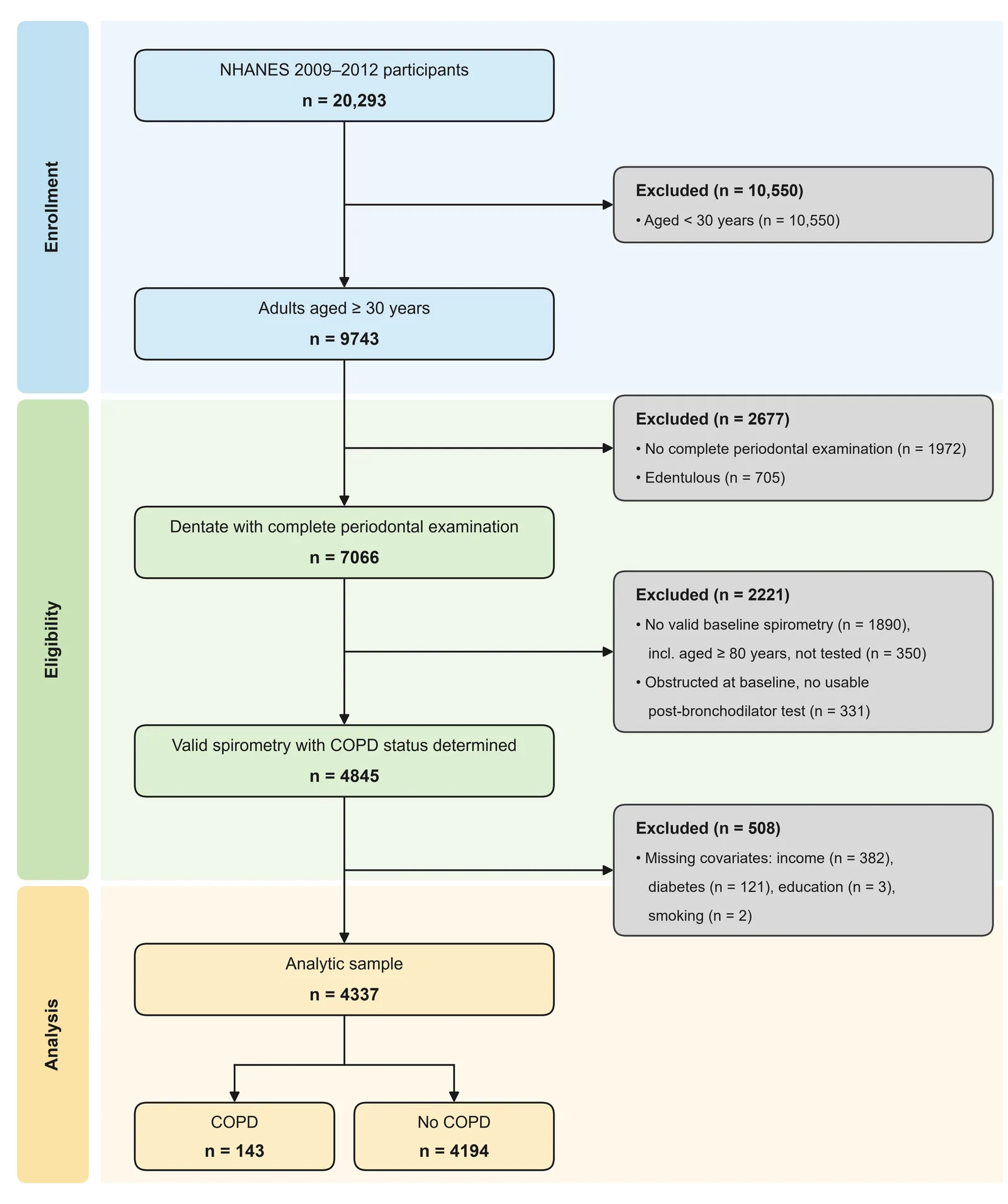
Flow diagram of participant selection, NHANES 2009–2012. Adults aged 80 years and older did not undergo spirometry in NHANES and were therefore excluded at the spirometry step. COPD: post-bronchodilator FEV1/FVC below the lower limit of normal.

This analysis included 4337 US adults aged 30 years and older from the 2009–2012 NHANES who underwent a full-mouth periodontal examination [13] and spirometry. Because NHANES did not perform spirometry in adults aged 80 years and older, no participants in this age group were included in the analytic sample. Examinations were performed by trained and calibrated dental professionals in mobile examination centers (MECs) using standardized NHANES examination procedures [21]. Eligibility for inclusion required a complete periodontal examination (OHDPDSTS = 1), at least one tooth measured, complete information on periodontal measures, a valid baseline spirometry test with a determinable post-bronchodilator COPD status, and covariates used in the regression analyses. Participants with missing data were excluded using listwise deletion. The number of participants excluded at each step is shown in Figure 1, and the extent of missing data for each variable is shown in Table S2.

### 2.3. Definition and Outcome

The primary outcome of interest (dependent variable) was periodontitis, classified as a binary variable (0 = no periodontitis, 1 = periodontitis). Periodontitis status was determined using data from the full-mouth periodontal examination and defined according to the 2012 Centers for Disease Control and Prevention/American Academy of Periodontology (CDC/AAP) case definitions for population-based surveillance of periodontitis [22]. Clinical attachment loss (CAL) and probing depth (PD) were measured at four interproximal sites per tooth (distofacial, mesiofacial, distolingual, and mesiolingual) on all teeth except third molars (teeth 2–15 and 18–31); sites that could not be assessed were treated as missing. Severe periodontitis was defined as ≥2 interproximal sites with CAL ≥ 6 mm (not on the same tooth) and ≥1 interproximal site with PD ≥ 5 mm; moderate periodontitis as ≥2 interproximal sites with CAL ≥ 4 mm (not on the same tooth) or ≥2 interproximal sites with PD ≥ 5 mm (not on the same tooth); and mild periodontitis as ≥2 interproximal sites with CAL ≥ 3 mm and ≥2 interproximal sites with PD ≥ 4 mm (not on the same tooth) or one site with PD ≥ 5 mm. Criteria requiring two or more sites were applied to sites on different teeth. Participants meeting the criteria for mild, moderate, or severe periodontitis were classified as having periodontitis. This surveillance definition differs from the 2018 European Federation of Periodontology (EFP)/AAP staging and grading framework, which is intended for the clinical classification of individual patients [23,24]. These standard definitions are based on clinical measures of periodontal attachment loss and probing depth collected during the NHANES examination and are widely used to assess periodontitis prevalence in population-based studies [22].

### 2.4. Independent Variables

The primary independent variable of interest was chronic obstructive pulmonary disease (COPD). COPD status was determined by spirometry performed in the NHANES mobile examination centers. Participants aged 6–79 years were eligible for baseline (pre-bronchodilator) spirometry, and participants with airflow obstruction at baseline (ratio of forced expiratory volume in 1 s to forced vital capacity [FEV1/FVC] below the lower limit of normal or below 0.70) were retested after inhaling a bronchodilator (albuterol; post-bronchodilator spirometry). Baseline tests were required to be complete (SPXNSTAT = 1), with forced expiratory volume in 1 s (FEV1; SPXNFEV1) and forced vital capacity (FVC; SPXNFVC) quality grades A or B (SPXNQFV1, SPXNQFVC); post-bronchodilator tests (SPXBFEV1, SPXBFVC) were required to be complete (SPXBSTAT = 1) with FEV1 and FVC quality grades A–C (SPXBQFV1, SPXBQFVC). For each participant, the lower limit of normal (LLN) of the FEV1/FVC ratio was calculated from the NHANES III reference equations of Hankinson et al. [25] using age, sex, and race/ethnicity (FEV1/FVC LLN = b0 + b1 × age, with sex- and race-specific coefficients). Race-specific equations were applied to non-Hispanic White, non-Hispanic Black, and Mexican American participants; the Caucasian equations were applied to other Hispanic and other race participants, for whom no specific equations are available. Consistent with the requirement of persistent airflow limitation [17], COPD was defined as a post-bronchodilator FEV1/FVC ratio below the LLN, as recommended by European Respiratory Society/American Thoracic Society (ERS/ATS) interpretive standards [18] and previously applied to NHANES spirometry [26]. Participants without obstruction at baseline were classified as not having COPD, and participants with baseline obstruction but no usable post-bronchodilator test were excluded. The variables and coding rules are listed in Table S1. To describe disease severity, participants with a post-bronchodilator FEV1/FVC < 0.70 (the Global Initiative for Chronic Obstructive Lung Disease [GOLD] spirometric criterion; *n* = 179) were classified by GOLD grade (GOLD 1: FEV1 ≥ 80% predicted; GOLD 2: 50–79%; GOLD 3: 30–49%; GOLD 4: < 30%) [17].

Several covariates were included as potential confounders based on prior epidemiologic literature examining periodontal disease and systemic conditions. These included diabetes status, cigarette smoking status, family income, educational attainment, sex, race/ethnicity, and age.

Diabetes status was classified as yes or no based on self-reported physician diagnosis; participants reporting borderline diabetes were treated as missing and excluded from the analysis. Cigarette smoking status was categorized as never smoker, former smoker, or current smoker. Never smokers were defined as individuals who reported smoking fewer than 100 cigarettes in their lifetime. Former smokers were those who reported smoking at least 100 cigarettes but did not currently smoke, while current smokers reported smoking every day or some days [27]. This classification, based on the threshold of 100 lifetime cigarettes, is the standard definition of smoking status used in US national health surveys and has been widely applied in previous population-based studies [27,28].

Educational attainment was dichotomized as high school or less and some college or above. Family income was categorized using the federal poverty level (FPL), dichotomized as <200% FPL and ≥200% FPL. Sex was categorized as male or female. Race/ethnicity was categorized as non-Hispanic White, non-Hispanic Black, Hispanic, and other races. Age was dichotomized as <65 years and ≥65 years because age 65 marks eligibility for Medicare, which affects access to medical care, including COPD diagnosis and treatment, and coincides with the loss of employer-based dental insurance for many adults, as routine dental care is not covered by traditional Medicare [29]. Oral hygiene was assessed as the number of days per week of dental floss use (OHQ870; none, 1–6 days, or daily).

### 2.5. Statistical Analysis

Descriptive statistics (unweighted counts and survey-weighted percentages) were conducted to summarize participant characteristics and the prevalence of periodontitis. The association between spirometry-defined COPD and periodontitis was examined using survey-weighted multiple logistic regression models. Adjusted odds ratios (aORs) and 95% confidence intervals (CIs) were reported. The final model controlled for diabetes status, cigarette smoking status, family income, educational attainment, sex, race/ethnicity, and age. All statistical analyses accounted for the complex NHANES design by incorporating the combined examination weights (WTMEC2YR/2; the two cycles were pooled and the weights divided by two according to National Center for Health Statistics (NCHS) analytic guidelines [20]), strata (SDMVSTRA; 29 strata), and primary sampling units (SDMVPSU; 62 PSUs) using Taylor series linearization, with the analytic sample analyzed as a subpopulation of the full design (33 design degrees of freedom). Confidence intervals (95%) and *p*-values for regression coefficients were based on the t distribution with 22 residual degrees of freedom. Survey weights and design variables were applied in all analyses to account for unequal selection probabilities and sampling structure. Analyses were conducted in Python 3.12: point estimates were obtained from survey-weighted logistic regression models fitted with statsmodels (version 0.14), and design-based variances were estimated by Taylor series linearization using custom code. All estimates were verified in R Statistical Software (version 4.3.3; Core R Team 2025) using the survey package (*svyglm* with a quasibinomial family), which gave identical results. Statistical significance was defined as a two-sided *p*-value < 0.05. Additional analyses examined the association by GOLD. In a sensitivity analysis, flossing frequency was added to the main model to assess whether accounting for oral hygiene changed the association between COPD and periodontitis. This study was reported in accordance with the Strengthening the Reporting of Observational Studies in Epidemiology (STROBE) guidelines, and the completed STROBE checklist is provided in the Supplementary Materials.

## 3. Results

### 3.1. Sample Characteristics

The analytic sample included 4337 adults aged 30 years and older who completed a full-mouth periodontal examination and valid spirometry (Figure 1; Table 1). Overall, 3.4% (95% confidence interval [CI] 2.6–4.2) of adults had post-bronchodilator spirometry-defined chronic obstructive pulmonary disease (COPD). Participants excluded because of missing or unusable spirometry were older and had a higher prevalence of periodontitis than those included (Table S2). The mean age of participants was approximately 49 years, and slightly more than half of the sample was female. Most participants were non-Hispanic White (weighted 70.7%). Overall, 7.8% reported diagnosed diabetes. In total, 57.9% were never smokers, 25.4% former smokers, and 16.6% current smokers. Slightly more than one-quarter of participants had household incomes below 200% of the federal poverty level (FPL), and 33.3% had a high school education or less.

Bivariate analysis (Table 1) showed that the overall prevalence of periodontitis among US adults was 40.2% (95% CI 36.8–43.6). The mean age of participants was 48.9 years (standard deviation [SD] = 12.6). Periodontitis prevalence was substantially higher among individuals with COPD (62.6%) than those without COPD (39.4%). Participants with diabetes also had a higher prevalence of periodontitis (58.8%) than those without diabetes (38.6%). Socioeconomic differences were observed, as individuals with household income below 200% of the federal poverty level had higher prevalence of periodontitis (55.1%) than those with income ≥ 200% FPL (34.5%). Similarly, participants with high school education or less had a higher prevalence (55.4%) than those with some college or above (32.6%).

Sex differences were present, with males having a higher prevalence of periodontitis (48.9%) than females (32.0%). Racial and ethnic disparities were also observed, with Hispanic (56.6%) and Black (53.8%) participants showing higher prevalence than White participants (34.7%), while participants categorized as other races had a prevalence of 46.3%.

Age-related differences were evident, as adults aged ≥65 years had higher prevalence of periodontitis (63.5%) than those younger than 65 years (37.4%). Cigarette smoking status was also associated with periodontal disease, with current smokers having the highest prevalence (60.8%), followed by former smokers (44.6%), and never smokers (32.3%).

### 3.2. Multivariable Logistic Regression Analysis

Multiple logistic regression analysis (Table 2) indicated that adults with post-bronchodilator spirometry-defined COPD had higher odds (adjusted odds ratio [aOR] = 1.99; 95% CI 1.16–3.41) of having periodontitis than those without COPD, after adjusting for diabetes, smoking status, income, education, sex, race, and age. Diabetes was also significantly associated with periodontal disease, with participants reporting diabetes having higher odds (aOR = 1.65; 95% CI 1.15–2.36) of periodontitis than those without diabetes.

Participants with income below 200% of the federal poverty level had higher odds (aOR = 1.57; 95% CI 1.25–1.97) of periodontitis than those with income ≥ 200% FPL. Individuals with at least some college education had lower odds of periodontitis (aOR = 0.58; 95% CI 0.50–0.67) than participants with a high school education or less. Females had lower odds of periodontitis than males (aOR = 0.49; 95% CI 0.44–0.55). Racial and ethnic differences were also observed, with Black (aOR = 2.17; 95% CI 1.57–3.01), Hispanic (aOR = 2.16; 95% CI 1.69–2.74), and other racial groups (aOR = 1.81; 95% CI 1.26–2.61) having higher odds of periodontitis than White participants. Adults aged ≥65 years had higher odds of periodontitis than those younger than 65 years (aOR = 3.65; 95% CI 2.59–5.15). Smoking status remained significantly associated with periodontal disease, with former smokers (aOR = 1.58; 95% CI 1.32–1.89) and current smokers (aOR = 2.84; 95% CI 2.24–3.61) having higher odds of periodontitis than never smokers.

### 3.3. Additional Analyses

Using the Global Initiative for Chronic Obstructive Lung Disease (GOLD) grade, the aOR for periodontitis was 2.13 (95% CI 1.05–4.29) for GOLD 1 (*n* = 114) and 1.72 (95% CI 0.82–3.62) for GOLD 2–4 (*n* = 65; only four participants were classified as GOLD 3 and none as GOLD 4) (Table S3).

Further adjustment for flossing frequency did not change the estimate (aOR = 2.00; 95% CI 1.15–3.49; Table S4). For comparison, the results of the original analysis using self-reported COPD (National Health and Nutrition Examination Survey [NHANES] 2013–2014; *n* = 3221) are shown in Tables S5 and S6.

## 4. Discussion

This study evaluated the association between chronic obstructive pulmonary disease (COPD) and periodontitis among US adults using National Health and Nutrition Examination Survey (NHANES) data. Findings indicated that adults with post-bronchodilator spirometry-defined COPD had higher odds of periodontitis (adjusted odds ratio [aOR] = 1.99, 95% confidence interval [CI]: 1.16–3.41) after adjusting for additional confounders. Because COPD was defined by spirometry rather than self-report, the analysis also captured adults with undiagnosed disease, and the use of post-bronchodilator spirometry restricted the exposure to persistent airflow limitation, as required for a clinical diagnosis of COPD. This finding is consistent with the study hypothesis.

Studies conducted in other countries have reported similar associations. However, these studies differed from the present study in their definitions of COPD and periodontal disease, the populations examined, and the factors included in their analyses. In a population-based study in Spain that used national health interview survey data from adults aged 40 years or older, in which COPD was self-reported and periodontal disease was defined by self-reported bleeding gums or tooth mobility, adults with COPD had 21% higher odds of periodontal disease (odds ratio [OR] = 1.21; 95% CI: 1.12–1.30) [15]. That study relied on survey data rather than spirometry and clinical periodontal examination, yet the direction of the association was the same as in the present study.

In Korean adults aged 40 years and older (Korea National Health and Nutrition Examination Survey [KNHANES] 2013–2015), in whom COPD was identified by pulmonary function testing, periodontal status measured with the Community Periodontal Index was associated with COPD in unadjusted analyses, but after adjustment for demographic, socioeconomic, and oral health factors, COPD was associated with tooth loss (OR = 1.32; 95% CI: 1.20–1.53) rather than with periodontal status [30]. Tooth loss and periodontitis are different outcomes, and that study examined COPD as the outcome, so its findings are not directly comparable with the present results.

Our findings are consistent with those of Zhang et al., who analyzed NHANES 2009–2014 and reported a bidirectional association between periodontitis and COPD, defined by a fixed ratio of forced expiratory volume in 1 s to forced vital capacity (FEV1/FVC) below 0.70 in 2009–2012 and by self-report in 2013–2014 [16]. The present study extends that work in several ways. First, COPD was defined as persistent airflow limitation after bronchodilation, using age-, sex-, and race/ethnicity-specific lower limits of normal rather than a fixed ratio, which misclassifies airflow limitation by age [18]. Second, COPD was examined as the exposure, complementing analyses of periodontitis as a risk factor for COPD. Third, we examined the association by Global Initiative for Chronic Obstructive Lung Disease (GOLD) grade to assess whether it differed across levels of airflow limitation, and further adjusted for flossing frequency to assess whether accounting for oral hygiene changed the association.

In the present study, COPD remained associated with periodontitis after adjustment for shared risk factors. This finding is consistent with a systematic review and meta-analysis of 12 studies, which reported higher odds of periodontitis among people with COPD (OR = 1.28; 95% CI 1.16–1.42) [31]. Several biological mechanisms are plausible based on the shared inflammatory and immune pathways, including the Janus Kinase (JAK) and Signal Transducer and Activator of Transcription (STAT) pathway [32]. In addition, neutrophils are the first-appearing inflammatory cells in gingivitis and have been found to heavily accumulate in periodontitis [3]. Cytokines have an important function in conducting inflammation in a range of inflammatory diseases, and the production of cytokines is an indicator of chronic inflammation in COPD [32]. Roles of neutrophils, cytokines, and oxidative stress are triggered in the activated pathways; specifically, oral pathogens influence lung inflammation via aspiration and microbial colonization [6]. These mechanisms are plausible explanations based on previous studies; however, the present analysis did not measure inflammatory markers or oral and airway microbiota and therefore cannot determine whether these pathways explain the observed association.

Furthermore, review articles have proposed that periodontal treatment might reduce COPD exacerbations and improve respiratory outcomes [6,7]; this hypothesis could not be evaluated in the present cross-sectional study.

A strength of this study is the use of NHANES, which is designed to be representative of the US civilian, non-institutionalized population, together with clinically measured COPD (spirometry) and periodontitis (full-mouth examination) in the same participants [19]. However, the analytic sample was restricted to dentate adults with a complete periodontal examination, usable spirometry, and complete covariate data, and these participants were younger and had a lower prevalence of periodontitis than those who were excluded (Table S2); the findings may therefore not generalize to all US adults, particularly older, edentulous, or less healthy adults. Studies in other populations have reported similar associations [6,31]; this agreement provides useful context but does not by itself establish the validity or generalizability of the present results. Collectively, these findings support an association between COPD and periodontitis and emphasize the importance of future longitudinal research to better understand the biological and inflammatory mechanisms linking these chronic conditions.

Limitations of this study were exemplified in multiple areas. Because COPD and periodontitis were measured at the same time, the cross-sectional design cannot establish whether COPD preceded periodontitis or vice versa; further analysis of the same cross-sectional data cannot resolve this, and longitudinal data are needed to examine the temporal relationship. The association may also be bidirectional: periodontal pathogens may contribute to airway inflammation through aspiration, while systemic inflammation and immune dysregulation in COPD may aggravate periodontal destruction [16]. Post-bronchodilator testing was performed only in participants with baseline obstruction, and participants with baseline obstruction but no usable post-bronchodilator test (approximately 5% of eligible participants) were excluded; these participants were older and had a higher prevalence of periodontitis, which may have led to an underestimate of COPD prevalence and biased the association. Smoking status was self-reported and classified as never, former, or current smoker; although this definition does not fully capture smoking intensity or duration and may be affected by reporting error, it is a widely used and well-established definition in population-based research [27,28]. Residual confounding by smoking cannot be fully excluded. Age was categorized at 65 years, so residual confounding by age cannot be fully excluded. Although flossing was examined, other factors such as dietary intake, dental care use, and medication use could not be fully assessed. The limitations of measurement highlight the lack of periodontitis grading beyond a binary classification of yes or no. With 143 participants with COPD, the precision of the estimates remained moderate. Few participants had severe or very severe airflow limitation (GOLD 3–4); therefore, associations with severe COPD could not be evaluated. Approximately 27% of otherwise eligible participants lacked a valid baseline spirometry test, including all participants aged 80 years and older, who were not tested; these participants were older and had a higher prevalence of periodontitis, so the complete-case approach may have introduced selection bias. Because the periodontal examination was limited to dentate adults aged 30 years and older and spirometry to adults younger than 80 years, no participants aged 80 years or older were included, and the findings should not be generalized to edentulous adults or to adults aged 80 years and older. The Hankinson equations do not include other Hispanic and other race groups, for whom the Caucasian equations were applied, and race-neutral reference equations were not examined. Finally, 2009–2012 are the most recent NHANES cycles with both spirometry and periodontal examinations; the data are therefore more than a decade old and may not reflect current patterns.

The public health and clinical implications of this study are important given the connection between COPD and periodontitis, as well as the broader relationship that medical and dental care can share to improve the quality of life of a patient with chronic respiratory disease. Patients identified with both conditions could be provided holistic preventive care through the collective advice of pulmonologists, primary care physicians, and dentists. Referrals and periodontal assessments for patients with COPD could help medical and dental providers identify oral inflammation earlier. Previous studies have suggested that periodontal treatment might reduce respiratory exacerbations and hospitalizations [6,7]; however, whether improving oral health affects COPD outcomes requires evaluation in longitudinal and interventional studies. Furthermore, given the high prevalence of periodontitis among current (60.8%) and former smokers (44.6%), promoting smoking abstinence and counseling must remain an aspect of dental and medical visits. From a public health standpoint, susceptible populations, including older adults aged 65 and above and low-income and lower education individuals, need to be given access to preemptive and restorative dental services to help curb oral health disparities, thus improving general health outcomes [29]. Many individuals who lack dental benefits through Medicare tend to be older adults, restricted by limited dental insurance coverage according to national data from the Centers for Disease Control and Prevention (CDC) [29]. Addressing the burden of COPD and periodontitis in high-risk populations would be a positive result from public health policies that encourage cooperation and integration between medical and dental care.

## 5. Conclusions

This study found that post-bronchodilator spirometry-defined chronic obstructive pulmonary disease (COPD) was associated with significantly higher odds of periodontitis among dentate US adults aged 30–79 years in the National Health and Nutrition Examination Survey (NHANES) 2009–2012 analytic sample. Diabetes, smoking, lower income, and older age were also associated with higher odds of periodontal disease, while higher education and female sex were associated with lower odds. These findings support an association between chronic respiratory disease and oral health. Further longitudinal studies are needed to better understand the biological pathways linking COPD and periodontitis.

## Figures and Tables

**Table 1:** Prevalence of periodontal disease among US adults aged 30–79 years by participant characteristics; NHANES 2009–2012 (post-bronchodilator spirometry-defined COPD).

Variable	*n* (Unweighted)	Weighted Percentage (95% CI)	Periodontal Disease, Weighted Percentage (95% CI)
Overall	4337	100%	40.2 (36.8–43.6)
Age in years (SD)	48.9 ± 12.6	48.4 ± 12.0	
COPD			
No	4194	96.6 (95.8–97.4)	39.4 (36.0–42.8)
Yes	143	3.4 (2.6–4.2)	62.6 (52.3–73.0)
Diabetes			
No	3888	92.2 (91.0–93.4)	38.6 (35.2–42.1)
Yes	449	7.8 (6.6–9.0)	58.8 (51.1–66.5)
Family income level			
≥200% FPL	2522	72.2 (69.3–75.2)	34.5 (30.4–38.5)
<200% FPL	1815	27.8 (24.8–30.7)	55.1 (51.7–58.4)
Education			
High school or less	1811	33.3 (30.0–36.6)	55.4 (51.6–59.3)
Some college or above	2526	66.7 (63.4–70.0)	32.6 (29.2–35.9)
Sex			
Male	2137	48.5 (46.5–50.5)	48.9 (45.1–52.7)
Female	2200	51.5 (49.5–53.5)	32.0 (28.6–35.4)
Race			
White	1918	70.7 (66.1–75.3)	34.7 (30.4–38.9)
Black	872	9.8 (7.7–12.0)	53.8 (47.9–59.8)
Hispanic	1111	13.3 (9.7–16.9)	56.6 (52.9–60.4)
Others	436	6.2 (4.9–7.5)	46.3 (38.7–54.0)
Age groups (in years)			
<65	3761	89.2 (87.7–90.7)	37.4 (33.9–40.8)
≥65	576	10.8 (9.3–12.3)	63.5 (56.6–70.4)
Cigarette smoking status			
Never smoker	2469	57.9 (55.4–60.4)	32.3 (28.9–35.8)
Former smoker	1046	25.4 (22.7–28.2)	44.6 (39.6–49.6)
Current smoker	822	16.6 (15.0–18.3)	60.8 (56.5–65.1)

Values are unweighted numbers (*n*) and survey-weighted percentages (WTMEC2YR/2). COPD: post-bronchodilator FEV1/FVC below the lower limit of normal (Hankinson/NHANES III equations). Periodontal disease: 2012 CDC/AAP case definition (mild, moderate, or severe). Age: unweighted and weighted mean ± SD. FPL: federal poverty level.

**Table 2:** Multiple logistic regression analysis of the presence of periodontal disease among US adults aged 30–79 years; NHANES 2009–2012 (post-bronchodilator spirometry-defined COPD).

Variable	Adjusted Odds Ratio, Survey-Weighted (95% CI)	*p* Value
COPD		
No	Reference	
Yes	1.99 (1.16–3.41)	0.015
Diabetes		
No	Reference	
Yes	1.65 (1.15–2.36)	0.009
Family income level		
≥200% FPL	Reference	
<200% FPL	1.57 (1.25–1.97)	<0.001
Education		
High school or less	Reference	
Some college or above	0.58 (0.50–0.67)	<0.001
Sex		
Male	Reference	
Female	0.49 (0.44–0.55)	<0.001
Race		
White	Reference	
Black	2.17 (1.57–3.01)	<0.001
Hispanic	2.16 (1.69–2.74)	<0.001
Others	1.81 (1.26–2.61)	0.003
Age groups (in years)		
<65	Reference	
≥65	3.65 (2.59–5.15)	<0.001
Cigarette smoking status		
Never smoker	Reference	
Former smoker	1.58 (1.32–1.89)	<0.001
Current smoker	2.84 (2.24–3.61)	<0.001

Survey-weighted logistic regression including all listed variables; *n* = 4337 (143 with COPD). COPD: post-bronchodilator FEV1/FVC below the lower limit of normal (Hankinson/NHANES III equations). 95% CIs and *p* values are based on the t distribution with 22 residual degrees of freedom. FPL: federal poverty level.

## Data Availability

The NHANES data that support the findings of the present study are publicly available at the CDC website [https://wwwn.cdc.gov/nchs/nhanes/Default.aspx, accessed on 15 March 2025]. The analysis code is available from the corresponding author upon reasonable request.

## Supplementary Materials

The following supporting information can be downloaded at https://www.mdpi.com/article/10.3390/healthcare14213727/s1, Table S1: NHANES variables and coding; Table S2: Missing data and characteristics of included and excluded participants; Table S3: COPD by GOLD grade; Table S4: Additional analyses of smoking, age, and oral hygiene; Table S5: Prevalence of periodontal disease by participant characteristics using self-reported COPD, NHANES 2013–2014; Table S6: Multiple logistic regression analysis using self-reported COPD, NHANES 2013–2014; Table S7: STROBE checklist for cross-sectional studies.
