# Supplementary material for: Exploring the Relationship Between Spirometry-Defined COPD and Periodontitis Among US Adults
Source: Healthcare (Basel). 2026 Nov 3;14(21):3727. doi: 10.3390/healthcare14213727 (PMC13763273; doi:10.3390/healthcare14213727)
Supplement: Supplementary file 1 [file healthcare-14-03727-s001.zip › healthcare-4596476-supplementary.pdf]

Supplementary Materials

Exploring the Relationship Between Spirometry-Defined COPD and Periodontitis Among US Adults (NHANES 2009–2012)

Table S1. NHANES variables and coding.

Variable	NHANES variable(s)	Coding
Periodontitis (outcome)	OHXxxLAD/S/P/A, OHXxxPCD/S/P/A (teeth 2–15, 18–31); OHDPDSTS	CDC/AAP surveillance definition (Eke et al. 2012): mild, moderate or severe = 1; none = 0. Code 99 = missing. Complete exam (OHDPDSTS = 1), ≥ 1 tooth measured.
Baseline spirometry	SPXNSTAT, SPXNFEV1, SPXNFVC, SPXNQFV1, SPXNQFVC	Complete test (SPXNSTAT = 1) with FEV1 and FVC quality grades A or B.
Bronchodilator selection	SPDBRONC	1 = selected for post-bronchodilator testing (baseline obstruction); 2 = not selected.
Post-bronchodilator spirometry	SPXBSTAT, SPXBFEV1, SPXBFVC, SPXBQFV1, SPXBQFVC	Usable test: SPXBSTAT = 1 with FEV1 and FVC quality grades A–C.
Lower limit of normal	RIDAGEYR, RIAGENDR, RIDRETH1	Hankinson (NHANES III) FEV1/FVC equations (age, sex, race/ethnicity); Caucasian equations for other Hispanic and other race.
Predicted FEV1 (GOLD grade)	RIDAGEYR, RIAGENDR, RIDRETH1, BMXHT	Hankinson (NHANES III) FEV1 equations (age, sex, height, race/ethnicity).
COPD (exposure)	—	1 = post-bronchodilator FEV1/FVC < LLN; 0 = no baseline obstruction (FEV1/FVC ≥ LLN and ≥ 0.70) or post-bronchodilator FEV1/FVC ≥ LLN; missing = baseline obstruction without a usable post-bronchodilator test.
Diabetes	DIQ010	1 = yes; 2 = no; 3 (borderline), 7, 9 = missing.
Smoking status	SMQ020, SMQ040	Never (< 100 cigarettes); former (≥ 100, not smoking now); current (≥ 100, every day or some days).
Family income	INDFMPIR	< 2.0 = < 200% of the federal poverty level (FPL).
Education	DMDEDUC2	1–3 = high school or less; 4–5 = some college or above; 7, 9 = missing.
Sex	RIAGENDR	Male; female.
Race/ethnicity	RIDRETH1	3 = non-Hispanic White; 4 = non-Hispanic Black; 1–2 = Hispanic; 5 = other.
Age group	RIDAGEYR	< 65 vs ≥ 65 years (continuous age in sensitivity analyses).
Flossing	OHQ870	Days per week: 0 = none; 1–6 = some; 7 = daily; missing = not reported (separate category).
Survey design	WTMEC2YR, SDMVSTRA, SDMVPSU	Weight WTMEC2YR/2; 29 strata; 62 PSUs; subpopulation analysis; 33 design df.

Table S2. Missing data and characteristics of included and excluded participants.

Variable	n missing	% of eligible
Baseline spirometry not usable (FEV1/FVC grade A/B)	1890	26.7
Post-bronchodilator COPD status not determinable	331	4.7
Diabetes (DIQ010; including borderline)	173	2.4
Smoking status (SMQ020/SMQ040)	3	0.0
Family income (INDFMPIR)	618	8.7
Education (DMDEDUC2)	11	0.2

Eligible participants: aged 30 years and older, complete periodontal examination and dentate (n = 7066). Categories are not mutually exclusive.

Characteristic	Included	Excluded: no usable baseline spirometry	Excluded: no post-bronchodilator status
n (unweighted)	4337	1647	296
Age, weighted mean (years)	48.4	56.2	55.8
Periodontitis, %	40.2	57.6	64.4
Age ≥ 65 years, %	10.8	32.9	27.7
Current smoker, %	16.6	18.0	29.8
Diabetes, %	7.8	12.9	12.1
Income < 200% FPL, %	27.8	40.4	35.3

Among eligible participants with complete covariates. Percentages and means are survey-weighted.

Table S3. COPD by GOLD grade.

Group	n	Weighted % of sample	Periodontitis, weighted % (95% CI)
No obstruction (post-BD FEV1/FVC ≥ 0.70)	4158	95.4	39.1 (35.8 – 42.4)
GOLD 1 (FEV1 ≥ 80%)	114	3.1	61.4 (49.2 – 73.5)
GOLD 2 (50–79%)	61	1.4	62.8 (47.3 – 78.4)
GOLD 3 (30–49%)	4	0.1	n too small
GOLD 4 (< 30%)	0	0.0	n too small

GOLD grade (ref = no obstruction)	n cases	aOR (95% CI)	p value
COPD: GOLD 1	114	2.13 (1.05–4.29)	0.036
COPD: GOLD 2–4	65	1.72 (0.82–3.62)	0.143

GOLD grades were assigned to participants with a post-bronchodilator FEV1/FVC < 0.70 (the GOLD spirometric criterion; n = 179). GOLD 1: FEV1 ≥ 80% predicted; GOLD 2: 50–79%; GOLD 3: 30–49%; GOLD 4: < 30% (post-bronchodilator FEV1, Hankinson predicted). GOLD 2–4 combined because only four participants were GOLD 3 and none GOLD 4. Adjusted for the Table 2 covariates.

Table S4. Additional analyses of smoking, age, and oral hygiene.

Analysis	NHANES variables added (data file)	n	COPD cases	aOR (95% CI)	p value
**A. Primary model**					
Primary: post-BD FEV1/FVC < LLN (Table 2)	Table 2 covariates only (see Table S1)	4337	143	1.99 (1.16–3.41)	0.015
**B. Smoking and age**					
Continuous age (age + age^2)	RIDAGEYR (DEMO); replaces age ≥ 65	4337	143	1.69 (1.04–2.75)	0.036
Never-smokers only	SMQ020 (SMQ); restricted to < 100 cigarettes	2469	38	2.84 (1.10–7.28)	0.032
**C. Oral hygiene**					
+ flossing frequency	OHQ870 (OHQ)	4337	143	2.00 (1.15–3.49)	0.017

All models are survey-weighted logistic regressions adjusted for the Table 2 covariates unless stated. Flossing model: participants with missing flossing information (n = 45) were kept as a separate "not reported" category, so the model uses the same sample as Table 2. BD, bronchodilator; LLN, lower limit of normal. NHANES data files: DEMO, demographics; SMQ, smoking – cigarette use; OHQ, oral health questionnaire; RXQ_RX, prescription medications (2009–2010 and 2011–2012 versions of each file).

Table S5. Prevalence of periodontal disease by participant characteristics using self-reported COPD, NHANES 2013–2014.

	n (unweighted)	Weighted percentage (95% CI)	Periodontal disease, weighted percentage (95% CI)
Overall	3221	100%	34.6 (29.7–39.6)
Age in years (SD)	51.8 ± 14.3	51.0 ± 13.7	
COPD (self-reported)			
No	3128	96.9 (95.9–98.0)	33.6 (28.6–38.6)
Yes	93	3.1 (2.0–4.1)	68.0 (58.2–77.8)
Diabetes			
No	2780	88.8 (87.5–90.1)	32.5 (27.5–37.4)
Yes	441	11.2 (9.9–12.5)	52.0 (46.5–57.4)
Family income level			
≥ 200% FPL	1834	68.8 (63.2–74.3)	27.3 (23.4–31.2)
< 200% FPL	1387	31.2 (25.7–36.8)	50.8 (43.6–58.0)
Education			
High school or less	1303	33.3 (29.0–37.7)	51.9 (46.8–57.0)
Some college or above	1918	66.7 (62.3–71.0)	26.0 (21.8–30.2)
Sex			
Male	1560	49.1 (47.4–50.8)	40.6 (35.3–45.9)
Female	1661	50.9 (49.2–52.6)	28.9 (23.8–34.0)
Race			
White	1436	68.8 (62.4–75.2)	29.5 (24.4–34.7)
Black	627	10.4 (7.7–13.1)	50.4 (43.1–57.6)
Hispanic	682	13.1 (8.4–17.7)	47.9 (41.5–54.3)
Others	476	7.7 (6.0–9.4)	36.7 (29.0–44.5)
Age groups (in years)			
< 65	2526	81.6 (79.8–83.3)	32.2 (27.2–37.2)
≥ 65	695	18.4 (16.7–20.2)	45.3 (38.2–52.5)
Cigarette smoking status			
Never smoker	1801	56.2 (53.3–59.0)	26.2 (21.2–31.3)
Former smoker	820	26.2 (24.2–28.3)	40.8 (35.1–46.5)
Current smoker	600	17.6 (15.7–19.5)	52.2 (45.0–59.5)

Original analysis using self-reported, physician-diagnosed COPD (MCQ160O: "ever told you had COPD"), NHANES 2013–2014, adults aged ≥ 30 years with a complete periodontal examination and ≥ 1 tooth; weight WTMEC2YR. This reproduces the submitted analysis (reported n = 3228; reproduced n = 3221); the Black and Hispanic rows, which were transposed in the submitted Table 1, are shown correctly here. Periodontal disease: 2012 CDC/AAP case definition. Percentages are survey-weighted; 95% CIs are Wald intervals. FPL: federal poverty level. Age: unweighted and weighted mean ± SD.

Table S6. Multiple logistic regression analysis using self-reported COPD, NHANES 2013–2014.

	Adjusted odds ratio, survey-weighted (95% CI)	p value
COPD (self-reported)		
No	Reference	
Yes	2.57 (1.07–6.21)	0.041
Diabetes		
No	Reference	
Yes	1.80 (1.40–2.31)	0.003
Family income level		
≥ 200% FPL	Reference	
< 200% FPL	1.88 (1.32–2.68)	0.008
Education		
High school or less	Reference	
Some college or above	0.48 (0.38–0.60)	<0.001
Sex		
Male	Reference	
Female	0.55 (0.45–0.68)	0.002
Race		
White	Reference	
Black	2.24 (1.44–3.49)	0.007
Hispanic	1.89 (1.21–2.94)	0.016
Others	1.79 (1.06–3.00)	0.036
Age groups (in years)		
< 65	Reference	
≥ 65	2.02 (1.43–2.87)	0.005
Cigarette smoking status		
Never smoker	Reference	
Former smoker	1.71 (1.23–2.38)	0.011
Current smoker	2.63 (1.74–3.98)	0.003

Original analysis using self-reported, physician-diagnosed COPD (MCQ160O: "ever told you had COPD"), NHANES 2013–2014, adults aged ≥ 30 years with a complete periodontal examination and ≥ 1 tooth; weight WTMEC2YR. This reproduces the submitted analysis (reported n = 3228; reproduced n = 3221); the Black and Hispanic rows, which were transposed in the submitted Table 1, are shown correctly here. Periodontal disease: 2012 CDC/AAP case definition. Survey-weighted logistic regression including all listed variables; 95% CIs and p values are based on the t distribution with 4 residual degrees of freedom (one survey cycle). FPL: federal poverty level.

Table S7: STROBE Checklist— Completed Mapping with Page References

Item	Section	STROBE recommendation	Page(s)	Where addressed
1a	Title/Abstract	Indicate study design in title/abstract	p. 1	Abstract, Background: "This cross-sectional study analyzed…"; keyword "Cross-Sectional Studies".
1b	Title/Abstract	Informative and balanced abstract	p. 1	Structured abstract (Background, Methods, Results, Conclusions) with sample size, COPD and periodontitis definitions, and main estimate.
2	Introduction	Background/rationale	pp. 1–2	Introduction: burden of COPD and periodontitis, oral health in COPD, shared inflammatory mechanisms, previous studies and their limitations (self-reported COPD, fixed-ratio spirometry).
3	Introduction	Objectives/hypotheses	p. 2	End of Introduction: study aim and hypothesis ("We hypothesized that adults with spirometry-defined COPD would have higher odds of periodontitis…").
4	Methods	Key design elements early	pp. 1, 2–3	Abstract (cross-sectional design); Methods – Data Source.
5	Methods	Setting, locations, dates	pp. 2–3	Methods – Data Source (NHANES 2009–2010 and 2011–2012; in-home interviews and mobile examination centers).
6a	Methods	Eligibility criteria and participant selection	p. 3; Figure 1 (p. 3)	Methods – Study Population (adults aged ≥30 years with a full-mouth periodontal examination, valid spirometry and complete covariates); Figure 1.
6b	Methods	Matching (if applicable)	N/A	Not a matched study.
7	Methods	Definitions of outcomes/exposures/confounders	pp. 4–5	Methods – Definition and Outcome (2012 CDC/AAP periodontitis); Independent Variables (post-bronchodilator FEV1/FVC < LLN; GOLD grades); covariates (diabetes, smoking, income, education, sex, race/ethnicity, age; flossing).
8	Methods	Data sources/measurement	pp. 2–5	Methods – NHANES examination procedures, spirometry quality criteria, Hankinson reference equations, covariate definitions.
9	Methods	Address potential bias	pp. 5, 10–11	Statistical Analysis (survey design; adjustment for flossing); Discussion – Limitations (selection, misclassification, residual confounding).
10	Methods	Study size rationale	pp. 3, 6; Figure 1 (p. 3)	Study Population (all eligible NHANES 2009–2012 participants who met the inclusion criteria); Results – Sample Characteristics (n = 4337); Figure 1.
11	Methods	Handling of quantitative variables	pp. 4–5	Covariates: income < 200% vs ≥ 200% FPL; education; age < 65 vs ≥ 65 years with rationale (Medicare eligibility); flossing (none, 1–6 days, daily); GOLD grades.

12a	Methods	Statistical methods/confounding control	p. 5	Statistical Analysis: survey-weighted logistic regression adjusted for diabetes, smoking, income, education, sex, race/ethnicity and age; aORs with 95% CIs.
12b	Methods	Subgroups/interactions	p. 5	Statistical Analysis: additional analyses by GOLD grade.
12c	Methods	Missing data handling	p. 3	Study Population: complete-case analysis; exclusions shown in Figure 1.
12d	Methods	Sampling strategy/weights	p. 5	Statistical Analysis: weights (WTMEC2YR/2), strata (SDMVSTRA), PSUs (SDMVPSU), Taylor series linearization, subpopulation analysis.
12e	Methods	Sensitivity analyses	p. 5	Statistical Analysis: adjustment for flossing frequency.
13a	Results	Numbers at each stage	p. 6; Figure 1 (p. 3)	Results – Sample Characteristics; Figure 1 (numbers at each step).
13b	Results	Reasons for non-participation	p. 3; Figure 1 (p. 3)	Reasons for exclusion and numbers excluded at each step in Figure 1.
13c	Results	Flow diagram	p. 3	Figure 1 (flow diagram of participant selection).
14a	Results	Participant characteristics	p. 6; Table 1 (pp. 6–7)	Results – Sample Characteristics; Table 1 (unweighted counts and weighted percentages).
14b	Results	Missing data per variable	p. 3; Figure 1 (p. 3)	Study Population; numbers excluded for missing data at each step in Figure 1.
15	Results	Outcome events/summary measures	p. 7; Table 1 (pp. 6–7)	Periodontitis prevalence overall (40.2%) and by COPD status (62.6% vs 39.4%); Table 1.
16a	Results	Adjusted/unadjusted estimates with precision	pp. 7–8; Tables 1–2 (pp. 6–8)	Unadjusted prevalences (Table 1); adjusted odds ratios with 95% CIs and p-values (Table 2); confounders and their rationale in Methods (p. 5).
16b	Results	Category boundaries for categorized variables	p. 5; Table 1 (pp. 6–7)	Methods – covariates; Table 1 (age < 65 / ≥ 65; income < 200% / ≥ 200% FPL).
16c	Results	Absolute risk translation (if relevant)	N/A	Not applicable (cross-sectional prevalence study); absolute prevalences given in Table 1.
17	Results	Other analyses (subgroup/sensitivity)	pp. 8–9	Results – Additional analyses (GOLD grade; adjustment for flossing).
18	Discussion	Key results summary	p. 9	Discussion, first paragraph.
19	Discussion	Limitations and bias direction/magnitude	pp. 10–11	Limitations paragraph (cross-sectional design; post-bronchodilator testing and exclusions; self-reported smoking; residual confounding; number of COPD cases; selection bias; reference equations; age of data).
20	Discussion	Cautious interpretation	pp. 9–11	Discussion (comparison with previous studies; mechanisms not measured); Limitations (cross-sectional design cannot establish temporal order); Conclusion.

21	Discussion	Generalisability	pp. 2, 10–11	Data Source (nationally representative design); Discussion – strengths and generalizability; Limitations (dentate adults; no participants aged ≥ 80 years).
22	Other information	Funding/role of funders	p. 11	Funding: no external funding. NHANES data are publicly available from CDC/NCHS (Data Availability Statement, p. 12).

Page numbers refer to the published article.
